# Quasi-experimental studies in the *Journal of Korean Biological Nursing Science* (2011~2024): an evaluation based on the TREND guidelines

**DOI:** 10.7586/jkbns.25.068

**Published:** 2025-11-21

**Authors:** Mi-Kyoung Cho, Mi Young Kim

**Affiliations:** 1 Department of Nursing Science, College of Nursing, Chungbuk National University, Cheongju, Korea; 2 College of Nursing, Hanyang University, Seoul, Korea

**Keywords:** Non-randomized controlled trials as topic, Guideline adherence, Quality control, Nursing research

## Abstract

**Purpose:**

This study evaluated the reporting completeness of quasi-experimental studies published in the *Journal of Korean Biological Nursing Science* from 2011 to 2024 using the Transparent Reporting of Evaluations with Nonrandomized Designs (TREND) guidelines.

**Methods:**

A systematic search identified 91 quasi-experimental studies with human participants. Reporting completeness was assessed using the TREND guidelines, and conceptual mapping was conducted to examine alignment with Template for Intervention Description and Replication (TIDieR), Joanna Briggs Institute (JBI) Critical Appraisal Checklist, and Risk of Bias in Non-randomized Studies of Interventions, Version 2 (ROBINS-I V2) domains.

**Results:**

Most studies involved non-pharmacological interventions (e.g., education, counseling, and behavioral programs). While participant eligibility, intervention description, and statistical methods were generally well reported, key elements (e.g., protocol modifications, intervention fidelity, confounder handling, and adverse effects) were often missing. Mapping revealed overlaps between 18 TREND items and TIDieR, JBI, and ROBINS-I V2 domains, but areas related to external validity, contextual interpretation, and theoretical linkage were not mapped.

**Conclusion:**

Reporting quality was moderate. Systematic adherence to the TREND guidelines, supplemented by TIDieR, JBI, and ROBINS-I V2, is recommended to increase transparency, reproducibility, and the value of quasi-experimental evidence in nursing science.

## INTRODUCTION

### 1. Background

In the field of nursing in Korea, quasi-experimental research has been frequently conducted to evaluate interventions related to practice, education, and policy. At the journal level, the quality management of reporting such studies directly affects the credibility of research and its applicability in clinical practice.

In nursing research, when randomization is not feasible due to ethical or practical constraints, quasi-experimental designs are often utilized. A quasi-experimental design is positioned between the rigor of a true experimental design—which includes at least one control group and one experimental/intervention group with random assignment [1]—and the flexibility of observational studies [2]. Quasi-experimental designs offer a practical method for evaluating intervention effects in real-world clinical and community settings. However, due to the absence of randomization, there are threats to internal validity such as selection bias. Therefore, transparency in design, analysis, and reporting is paramount [3,4]. In nursing and public health intervention studies, the clear reporting of intervention content, implementation context, characteristics of the comparison group, and strategies for bias adjustment plays a crucial role in the interpretation and reproducibility of research findings [3,5]. Incomplete reporting can lead to misunderstandings about the actual effects and limitations of interventions, which in turn can negatively affect the design of subsequent studies and policy decisions [5,6].

In response to these concerns, Des Jarlais et al. [3] published the Transparent Reporting of Evaluations with Nonrandomized Designs (TREND) statement in 2004 with the aim of improving the completeness of reporting for nonrandomized intervention evaluation studies. The TREND statement provides a 22-item checklist covering essential elements of quasi-experimental and nonrandomized research, including study design, detailed descriptions of interventions and comparison conditions, measurement and analysis methods, and bias adjustment strategies, thereby promoting standardization of reporting [7]. Since its introduction, TREND has been widely cited as a benchmark for improving the reporting quality of nonrandomized evaluation studies [5,8].

The *Journal of Korean Biological Nursing Science* (JKBNS) is a leading domestic academic journal that covers the overall field of biological nursing research, particularly nursing interventions, physiological studies, and educational research. The reporting practices of quasi-experimental designs in this journal reflect the quality of the research as well as the quality of information provided to its readership. However, there has been limited systematic review regarding the extent to which quasi-experimental studies published in this journal adhere to reporting guidelines such as TREND. Therefore, this study aims to analyze quasi-experimental studies published in the JKBNS between 2011 and 2024 in accordance with international reporting guidelines.

The widely used TREND checklist (TREND statement), developed in 2004, was designed to improve the completeness of reporting in nonrandomized or quasi-experimental intervention studies. It emphasizes detailed reporting of interventions and comparison conditions, study design, and strategies for adjusting potential biases [3]. However, there has been increasing discussion in recent years about the scope, limitations, and need for updating the TREND guideline [5,8].

Meanwhile, one of the international efforts to improve inadequate reporting practices is the Template for Intervention Description and Replication (TIDieR) checklist. TIDieR was developed to strengthen the reproducibility and transparency of intervention descriptions by including elements such as “what”, “how”, “who”, and “when and how much”, with a particular focus on describing complex interventions [9]. Accordingly, this study seeks to compare and analyze these two reporting guidelines and propose the guideline deemed more suitable for future application in the field of nursing.

Thus, while reporting guidelines and critical appraisal tools serve different functions in ensuring the transparency and credibility of research reporting, there is a lack of studies that identify which items are vulnerable in quasi-experimental research and compare which guideline or tool can act complementarily. Moreover, in the case of domestic journals, there are limited instances of quantitative assessment and comparison of reporting quality.

Therefore, this study aimed to conduct a multi-layered evaluation of the current status of quasi-experimental study reporting and methodological quality by mapping the TREND checklist with tools such as TIDieR, the Joanna Briggs Institute (JBI) Critical Appraisal Checklist, and Cochrane’s Risk of Bias in Non-randomized Studies of Interventions, Version 2 (ROBINS-I V2). The goal was to provide specific insights to domestic nursing intervention researchers regarding which areas are vulnerable and how reporting and study design should be improved. This effort is expected not only to standardize reporting practices but also to reduce the risk of misattribution and misinterpretation when research findings are applied to actual clinical practice or policy. Ultimately, the results of this study will serve as foundational data to strengthen the quality management system of the JKBNS and explore future directions for the journal’s qualitative development.

### 2. Study aim

This study aimed to objectively evaluate the qualitative level of quasi-experimental studies published in the JKBNS between 2011 and 2024 by applying international reporting guidelines appropriate for each study design.

## METHODS

### 1. Study design

This study is a methodological review that analyzed the adherence to the TREND reporting guideline among quasi-experimental studies published in the JKBNS from 2011 to 2024.

### 2. Samples

**Figure 1.**
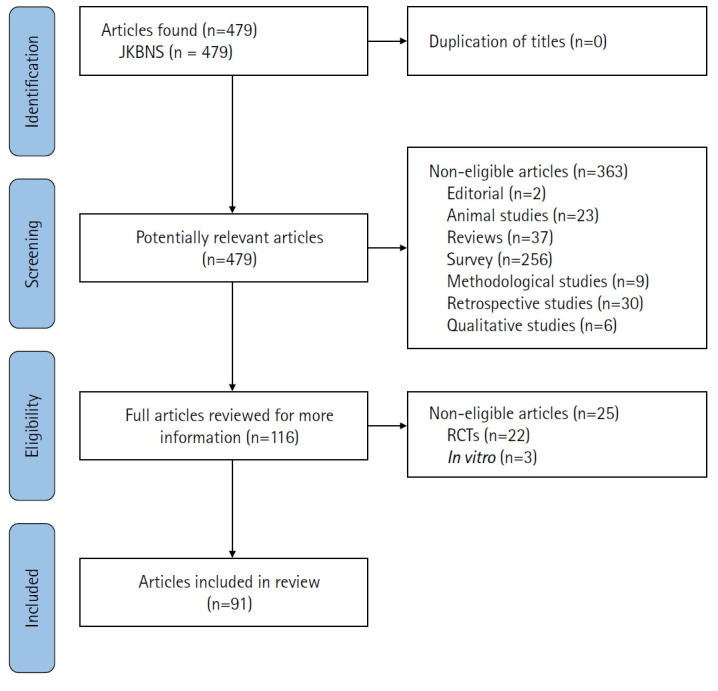
Flow chart of study selection. JKBNS = *Journal of Korean Biological Nursing Science*; RCTs = Randomized controlled trials.

The data for this study consisted of quasi-experimental pre-post studies published in the JKBNS between 2011 and 2024. The inclusion criteria were original research articles involving human participants that explicitly identified the study design as quasi-experimental in the title, abstract, or methods section. The exclusion criteria included editorials, animal studies, in vitro studies, review articles including meta-analyses, randomized controlled trials, survey studies, methodological studies, retrospective studies, and qualitative studies. Based on these criteria, a total of 91 articles were included in the final analysis (Figure 1, Appendix 1).

### 3. Instruments

In this study, the TREND checklist was employed to evaluate whether quasi-experimental studies adhered to established reporting standards. To map relevant components of the TREND checklist, additional evaluation tools were utilized, including the TIDieR checklist, the JBI Critical Appraisal Checklist for Quasi-Experimental Studies, and Cochrane’s ROBINS‑I V2.

#### 1) The TREND checklist

In this study, the quality of reporting in nonrandomized experimental studies was systematically assessed using the TREND checklist. Nonrandomized experimental designs are frequently employed in real-world clinical and community settings due to practical and ethical constraints, and they are particularly common in behavioral and public health intervention research. However, the absence of random allocation renders such studies more susceptible to various forms of bias, and inadequate reporting of intervention content and contextual factors can hinder the interpretation and generalizability of the findings. To address these limitations and enhance the completeness of reporting in studies utilizing nonrandomized designs, Des Jarlais et al. [3] (2004) developed the TREND checklist. This tool was adapted from the Consolidated Standards of Reporting Trials (CONSORT) guidelines and revised to reflect the structural features of nonrandomized study designs. The TREND checklist comprises 22 items in total, including 21 core items across five key sections—title and abstract, introduction, methods, results, and discussion—along with a single item of overall evidence. Most of the items include one or more sub-items, each designed to reflect essential elements of transparent reporting across the conventional structure of a scientific manuscript. Key items include participant eligibility criteria, descriptions of the intervention and comparison conditions, methods of assignment, unit of analysis, statistical techniques, and procedures for interpreting results. Particularly important for non-randomized trials, the checklist also addresses intervention fidelity, statistical adjustment for potential confounders, the use of post-hoc analyses, and the reporting of adverse or unintended outcomes [7,10].

Beyond the documentation of study outcomes, the TREND checklist emphasizes the reporting of the theoretical foundation of the intervention, the proposed causal pathway, feasibility of implementation, and participant compliance. Through this comprehensive approach, the tool aims to improve both the interpretability and replicability of nonrandomized intervention studies. In this study, each item in the TREND checklist is evaluated based on the extent to which the corresponding content is reported in the included articles. Reporting is scored as "fully reported" (1 point) when all required information is clearly presented, "partially reported" (0.5 points) when only part of the information is provided, and "not reported" (0 points) when the item is entirely omitted.

#### 2) TIDieR checklist

The TIDieR checklist is a reporting guideline developed to enhance the reproducibility and transparency of intervention research by promoting the detailed description of intervention components and delivery processes. Hoffmann et al. [9] proposed the TIDieR checklist as an extension of Item 5 of the CONSORT 2010 statement for randomized trials and Item 11 of the Standard Protocol Items: Recommendations for Interventional Trials 2013 guideline, which focuses on the description of interventions in study protocols. This tool was specifically designed for use with complex interventions or non-pharmacological treatments that often involve multiple interacting components. Rather than limiting its scope to outcome reporting, TIDieR encourages researchers to provide a comprehensive account of how the intervention was structured and implemented, thereby enabling clinicians and other researchers to understand and potentially replicate the intervention with fidelity [9,11,12]. The checklist comprises 12 items that cover essential elements such as the intervention’s name, theoretical rationale, materials and procedures used, characteristics of the providers, mode of delivery, setting, frequency and intensity of delivery, tailoring, modifications made during the study, and fidelity to the planned delivery. Each item is designed to address core questions related to who delivered the intervention, what was delivered, when, where, how, and to what extent. Notably, the checklist emphasizes reporting elements such as intervention fidelity, contextual factors, whether the intervention was tailored to individual or group needs, and any modifications made throughout the implementation process. This approach moves beyond basic reporting to facilitate a deeper understanding of the nature of the intervention. By doing so, TIDieR supports the assessment of both the internal validity and external applicability of intervention studies. It provides a practical foundation for real-world implementation and future research planning. Each item of the TIDieR checklist is assessed using a three-point scale [13]: “fully reported” (2 points), “partially reported” (1points), and “not reported” (0 points).

#### 3) JBI Critical Appraisal Checklist for Quasi‑Experimental Studies

The JBI Critical Appraisal Checklist for Quasi-Experimental Studies is a standardized tool developed to evaluate the methodological quality of intervention studies that do not use random allocation. This checklist is specifically designed to assess the risk of bias in quasi-experimental research by examining key aspects such as study design, implementation of the intervention, presence of a comparison group, reliability of outcome measurements, and appropriateness of statistical analyses [14]. The checklist comprises nine items, each reflecting a fundamental component of methodological rigor. The items address the following aspects: (1) whether a clear causal relationship is established with appropriate temporal sequencing between cause and effect, (2) whether the comparison groups are similar in their baseline characteristics, (3) whether participants across groups received comparable care aside from the intervention, (4) whether a control group was included, (5) whether outcomes were measured at multiple time points both before and after the intervention, (6) whether follow-up was complete, or, if not, whether the attrition and its potential impact were adequately explained, (7) whether outcomes were measured in the same way across all comparison groups, (8) whether the measurement tools used were valid and reliable, and (9) whether appropriate statistical analysis was conducted. Each item is evaluated using a categorical scale comprising four possible responses: “Yes”, “No”, “Unclear”, and “Not applicable”.

#### 4) ROBINS‑I V2

The Cochrane ROBINS-I V2 tool has been developed to systematically assess the risk of bias in non-randomized studies of interventions when estimating the effectiveness or harm of an intervention [15,16]. It covers study designs, including cohort, case-control, and quasi-randomized designs. It uses the internal validity standards of randomized controlled trials (RCTs) as a benchmark to evaluate structural sources of bias inherent in non‑randomized designs [16]. Compared to its previous version, ROBINS-I V2 refines its evaluation algorithms and signaling question framework, introduces a triage procedure, and incorporates considerations such as time-varying confounding and immortal time bias to enhance both sensitivity and applicability [16]. ROBINS‑I V2 is structured around seven bias domains: (1) confounding; (2) selection of participants; (3) classification of interventions; (4) deviations from intended interventions; (5) missing outcome data; (6) measurement of outcomes; and (7) selection of the reported result [16]. Each domain is assessed through pre‑specified signaling questions with response options including “strong yes”, “weak yes”, “weak no”, “strong no”, or “no information”. Moreover, it incorporates a triage stage to identify results with a critical risk of bias early in the assessment process. Judgments for each domain are classified into one of four levels: low, moderate, serious, or critical. An overall risk of bias is then assigned for each specific outcome by integrating these domain‑level judgments.

### 4. Data collections

Data were retrieved from the JKBNS official website archive using the keywords “quasi‑experimental”, “non-randomization”, and “experimental”. The search was conducted between July 21 and July 31, 2025. The identified articles were catalogued in an Excel spreadsheet containing author(s), publication year, title, and DOI. From August 1 to August 10, 2025, two independent reviewers (Cho & Kim) examined the titles, study design, and full texts of the retrieved articles. In the first screening stage, studies were selected whose titles included terms such as “experimental study”, “non-randomization”, “quasi*”, “experimental”, or “효과”. In the second stage, abstracts were reviewed to extract those explicitly describing “quasi‑experimental”, “experimental”, or similar designs. During the third stage, articles labeled in the methods section as “quasi‑experimental study” were further examined; those that included descriptions of random assignment in the full text were reclassified as RCTs and excluded. Disagreements over inclusions primarily concerned whether to include single‑group pre‑post studies or comparison studies; after discussion, it was agreed to include them.

### 5. Data analysis

Data analysis was conducted to systematically summarize the general characteristics and report the quality of the included quasi-experimental studies. It also aimed to compare the conceptual alignment and differences across reporting guideline tools for non-randomised studies. General study characteristics—such as author(s), year of publication, whether reporting guidelines were specified, study design type, participant characteristics, and sample size, intervention methods for experimental and control groups, type of intervention, and dependent variables—were extracted from the full texts and organized using a coding book in Excel. These characteristics were then summarized using frequencies and percentages. Reporting quality was assessed based on the 22 items of the TREND checklist. Each item was coded as “fully reported” (1 point), “partially reported” (0.5 points), or “not reported” (0 points), depending on the degree of adherence. Compliance rates for individual checklist items were calculated as frequencies and percentages. The total and mean scores for each article were used to evaluate the overall level of reporting. Additionally, a conceptual mapping was performed to compare the items in the TREND checklist with those in the TIDieR Checklist, the JBI Checklist for Quasi-Experimental Studies, and Cochrane’s ROBINS-I V2.

### 6. Ethical considerations

This review was conducted using previously published studies that involved human participants, without the collection of new data or direct involvement with research subjects. Therefore, this study was exempt from Institutional Review Board (IRB) oversight. In adherence to research ethics, all sources of data were properly cited. To reduce potential bias in the selection and evaluation process, two independent reviewers assessed the eligibility of the included articles, and any disagreements were resolved through consensus.

## RESULTS

### 1. Characteristics of quasi-experimental studies published in JKBNS (2011~2024)

This study analyzed the characteristics of 91 quasi-experimental studies published between 2011 and 2024 in the JKBNS. Many studies (n = 50) were published between 2011 and 2015, while only 13 studies were published from 2021 to 2024. Sixty-four studies (70.3%) reported IRB approval, and 24 studies (26.4%) received research funding. Regarding study design, 81 studies (89.0%) were a control group design. In sample size, 43 studies (47.2%) included fewer than 50 participants, and 41 studies (45.1%) had 50 to 99 participants. The most common participant characteristics were included adults and the elderly, aged 18 years or older (n = 39, 42.9%), and patient populations (n = 44, 48.3%). Most interventions applied to experimental groups were non-pharmacological interventions. Education-based interventions were the most common (n = 23, 25.3%), followed by exercise interventions (n = 9) and music therapy (n = 5). Regarding the duration of the interventions, 67 studies (73.5%) implemented programs lasting less than eight weeks. In the number of intervention sessions, 25 studies (27.5%) reported fewer than 24 sessions, while 48 studies did not report specific information on the number of sessions. Post-intervention measurements were most frequently conducted immediately after program completion (n = 77, 84.6%). A variety of outcome variables were assessed, including depression, anxiety, blood pressure, pain, self-efficacy, fatigue, and pulse rate, indicating that the studies evaluated the effects of interventions across both psychosocial and physiological dimensions (Table 1).

### 2. Evaluation of reporting levels in quasi-experimental studies using the TREND checklist

This study evaluated the extent to which items from the TREND checklist were reported in quasi-experimental studies. A review by individual checklist items revealed that the following were clearly reported in all 91 studies: 1. Title & abstract, 2. Background, 3. Participants, 4. Interventions, 5. Objectives, 6. Outcomes, 10. Unit of analysis, 11. Statistical methods, 13. Recruitment, 14. Baseline data, 16. Numbers analyzed, 17. Outcomes & estimation, 20. Interpretation, 21. Generalizability, and 22. Overall evidence.

Items ‘8. Assignment method’ and ‘15. Baseline equivalence’ were reported in 90 studies, and ‘9. Blinding’ was partially reported with only one study. Items ‘7. Sample size’ and ‘12. Participant flow’ were reported in 88 studies. Item ‘18. Ancillary analyses’ was reported in only one study, and Item ‘19. Adverse events’ were reported in two studies (Table 2).

### 3. Mapping the TREND checklist to TIDieR, JBI, and ROBINS-I V2 for quasi-experimental studies

Each item of the TREND checklist was mapped to corresponding components of three widely used frameworks in the context of non-randomized studies: the TIDieR checklist, the JBI Critical Appraisal Checklist, and Cochrane’s ROBINS-I V2 tool (Table 3). Among all TREND items, only one—“4. Methods: Interventions”—was found to align with all three tools, indicating its shared emphasis across reporting and quality assessment standards. In contrast, both the JBI and ROBINS-I V2 tools focus predominantly on the methodological rigor and validity of outcome reporting, with no explicit coverage of discussion-related items, which are included in the TREND checklist. In the mapping between the TREND and TIDieR checklists, “1. Title and abstract” corresponded with Item 1 of TIDieR, and “2. Introduction” aligned with Item 2. The TREND item “4. Methods: interventions” was broadly matched with TIDieR Items 3 to 12, which detail various aspects of intervention design, delivery, and implementation fidelity. For the JBI Critical Appraisal Checklist, “5. Methods: objectives” aligned with Item 1, while “2. Methods: participants” matched Item 2. “14. Results: baseline data” corresponded with Items 2 and 3. Additionally, “4. Methods: interventions” aligned with Item 4, and “6. Methods: outcomes” matched Items 5 to 8. The items “15. Results: baseline equivalence” and “16. Results: numbers analyzed” aligned with Items 5 and 8, respectively, while “17. Results: outcomes and estimation” corresponded with Items 7 and 8. Finally, “11. Methods: statistical methods” and “18. Results: ancillary analyses” both matched Item 9 of the JBI checklist.

Regarding the ROBINS-I V2 tool, the following domains were mapped: TREND items 2, 7, 11, 14, and 15 were linked to Domain 1 (Bias due to confounding). Item 3 corresponded to Domain 2 (Classification of interventions). Item 4 aligned with both Domain 3 (Selection of participants) and Domain 4 (Deviations from intended interventions). Item 12 was mapped to Domains 4 and 5 (Missing data). Items 5, 6, 8, and 9 were associated with Domain 6 (Measurement of outcomes), and items 10 and 17~19 corresponded with Domain 7 (Selection of the reported result).

## DISCUSSION

The quasi-experimental intervention studies analyzed in this study were generally evaluated as meeting many of the TREND criteria. In other words, from the title to the discussion sections, the overall structure was well established, indicating that most items were reported. However, several critical elements, particularly the reporting of adverse events, were found to be very limited.

Regarding adverse event reporting, previous research on clinical trials and intervention studies found that while 46% of published articles reported adverse events, as many as 95% were identified in unpublished sources. This suggests that when relying solely on published literature, approximately 64% of all adverse events could be omitted from analyses [17]. Such findings indicate that adverse events are often underreported in published research. In the studies analyzed for this research, even when reasons for participant dropout were reported, they were primarily attributed to external factors (e.g., symptom complaints) rather than serious adverse events caused by the intervention itself. This may be explained by the fact that nursing interventions are often non-pharmacological or primarily focused on education. Nonetheless, concerns remain that some actual harms may go unreported due to reproducibility issues or reporting limitations in clinical trials. The Cochrane Manual also emphasizes that reporting only favorable outcomes in research can lead to biased interpretations [18].

Therefore, efforts must be made to ensure that adverse events are consistently reported without omission. In addition, almost none of the studies reviewed reported blinding, with only one study explicitly mentioning it, while the rest did not. This lack of reporting is likely due to the inherent limitations of quasi-experimental designs, where randomization and blinding are often difficult to implement. In addition, the reporting rate for participant flow was low. Among the studies included in this research, there were 22 RCTs and 91 quasi-experimental studies, highlighting a notable imbalance. This difference reflects a limitation in terms of the rigor of the study design. Consequently, it is recommended that future research designs be structured more tightly and, where possible, evolve toward the level of rigor seen in RCTs. In addition, it is recommended to use the TREND checklist when reporting quasi-experimental studies to maintain rigor in reporting aspects other than randomization.

From a methodological perspective, minimizing bias and writing manuscripts in accordance with reporting guidelines can greatly enhance the reliability of future research. Although many studies in this research adhered to reporting guidelines, compliance alone does not fully guarantee internal validity. Rigorous experimental designs and bias-control strategies are also necessary to support the true effectiveness of interventions.

In this study, both reporting guidelines and quality appraisal tools were compared and analyzed. The TREND checklist offers comprehensiveness by requiring reporting across all sections, from the title to the discussion, whereas TIDieR focuses specifically on replication and intervention description. Recently, the use of TIDieR has been increasing, reflecting researchers’ growing need to transparently report “how the intervention was designed and implemented,” particularly in non-pharmacological intervention studies.

In practice, several international nursing journals explicitly require or recommend the use of the TIDieR checklist in their author guidelines. For instance, the Journal of Clinical Nursing and Nursing Open require authors to comply with the TIDieR checklist in conjunction with the CONSORT guidelines when submitting manuscripts [19,20]. Similarly, the International Journal of Nursing Sciences and International Journal of Nursing Studies Advances emphasize adherence to TIDieR to ensure reproducibility in intervention studies [21,22]. Furthermore, the Journal of Emergency Nursing strongly recommends following the TIDieR guidelines and checklist when submitting manuscripts focused on intervention development [23]. This trend indicates that TIDieR is gradually becoming an essential reporting standard in international journals, representing an evolving academic consensus aimed at enhancing the transparency and credibility of intervention reporting.

In contrast, many domestic nursing journals remain at the level of recommending general compliance with comprehensive reporting guidelines, such as those provided by the EQUATOR Network [24], without requiring the TIDieR checklist as a mandatory submission item. Therefore, to elevate the quality of intervention reporting by domestic researchers to meet international standards, it is necessary for domestic journals to gradually adopt TIDieR. This will ultimately improve the reproducibility of intervention studies and enhance their clinical applicability. Although there are various reporting guidelines and critical appraisal tools, some have pointed out that the mere publication of reporting guidelines does not automatically lead to improvements in reporting quality [25]. Therefore, it is also necessary to explore strategies for enhancing the quality of manuscripts through the effective application of reporting guidelines.

Ultimately, in applied disciplines such as nursing, it is important to strike a balance between the TREND approach, which requires comprehensive and detailed reporting from start to finish, and the TIDieR approach, which emphasizes the reproducibility of interventions. Strictly adhering to every single reporting item may impose a heavy burden on researchers, so a more practical alternative could be the gradual introduction of reporting guidelines focused on core intervention-related items. For example, certain items such as mid-trial stopping plans or documentation of fidelity changes may not be highly relevant to non-pharmacological intervention studies and could therefore be considered as optional reporting elements.

This study was limited to evaluating publications reported in JKBNS. If study designs or intervention details were not fully reported by the researchers, it may have been difficult to accurately assess the methodological quality. Furthermore, given that most interventions in nursing are non-pharmacological and have a low likelihood of causing adverse effects, the omission of adverse event reporting may lead to an underestimation of potential risks. Future studies could address this limitation by directly contacting researchers about selected studies or obtaining original materials (e.g., protocols, supplementary documents) to supplement missing information and then conducting a reassessment.

## CONCLUSION

This study explored the complementary use of reporting guidelines and critical appraisal tools in quasi-experimental research and examined the reporting quality of studies, focusing on domestic nursing journals. The findings revealed that quasi-experimental studies reported in JKBNS generally demonstrated a high level of reporting quality. The TREND reporting guideline contributed to enhancing the transparency and reproducibility of research; however, certain items were found to be more precisely evaluated using critical appraisal tools. Therefore, future studies should consider the combined use of various reporting guidelines and critical appraisal tools to further improve the reliability and validity of research.

## Figures and Tables

**Table 1.** Summary of Characteristics of Quasi-experimental Studies Published in JKBNS (2011~2024) (N = 91)

Characteristics		n	%
Publication year	2011~2015	50	54.9
	2016~2020	28	30.8
	2021~2024	13	14.3
IRB	No	27	29.7
	Yes	64	70.3
Fund	No	67	73.6
	Yes	24	26.4
Study design	One-group design	10	11.0
	Control group design	81	89.0
Number of participants	< 50	43	47.2
	50~99	41	45.1
	≥100	7	7.7
Age (years)	< 18	4	4.4
	≥ 18	14	15.4
	≥ 60	25	27.5
	Not reported	48	52.7
Sex	Both	79	86.8
	Men only	3	3.3
	Unclear	9	9.9
Participants	Nurse	10	11.0
	Students	12	13.2
	Patients	44	48.3
	Older adults	11	12.1
	Others	14	15.4
Intervention of the experimental group	Education	23	25.3
	Exercise	9	9.9
	Music	5	5.5
	Massage	3	3.3
	Others	51	56.0
Duration of intervention	< 8 weeks	66	72.5
	≥ 8 weeks	21	23.1
	Not reported	4	4.4
Number of sessions	< 24	25	27.5
	≥ 24	18	19.8
	Not reported	48	52.7
Measurement	Immediately	77	84.6
	Delayed	14	15.4
Outcome variables	Depression	11	12.1
	Blood pressure	7	7.7
	Pain	7	7.7
	Self-efficacy	7	7.7
	Anxiety	9	9.9
	Fatigue	5	5.5
	Pulse	5	5.5
	BMI	3	3.3
	Quality of life	3	3.3
	Others	34	37.3

JKBNS = *Journal of Korean Biological Nursing Science*; IRB = Institutional review board; BMI = Body mass index.

**Table 2.** Evaluation of Reporting Levels in Quasi-experimental Studies Using the TREND Checklist (N = 91)

TREND checklist items	Fully reported	Partially reported	Not reported
	n (%)		
1. Title & abstract	91 (100.0)	0 (0.0)	0 (0.0)
2. Background	91 (100.0)	0 (0.0)	0 (0.0)
3. Participants	91 (100.0)	0 (0.0)	0 (0.0)
4. Interventions	91 (100.0)	0 (0.0)	0 (0.0)
5. Objectives	91 (100.0)	0 (0.0)	0 (0.0)
6. Outcomes	91 (100.0)	0 (0.0)	0 (0.0)
7. Sample size	89 (97.8)	0 (0.0)	2 (2.2)
8. Assignment method	90 (98.9)	0 (0.0)	1 (1.1)
9. Blinding	0 (0.0)	1 (1.1)	90 (98.9)
10. Unit of analysis	91 (100.0)	0 (0.0)	0 (0.0)
11. Statistical methods	91 (100.0)	0 (0.0)	0 (0.0)
12. Participant flow	88 (96.7)	1 (1.1)	2 (2.2)
13. Recruitment	91 (100.0)	0 (0.0)	0 (0.0)
14. Baseline data	91 (100.0)	0 (0.0)	0 (0.0)
15. Baseline equivalence	90 (98.9)	0 (0.0)	1 (1.1)
16. Numbers analyzed	91 (100.0)	0 (0.0)	0 (0.0)
17. Outcomes & estimation	91 (100.0)	0 (0.0)	0 (0.0)
18. Ancillary analyses	1 (1.1)	0 (0.0)	90 (98.9)
19. Adverse events	2 (2.2)	0 (0.0)	89 (97.8)
20. Interpretation	91 (100.0)	0 (0.0)	0 (0.0)
21. Generalizability	91 (100.0)	0 (0.0)	0 (0.0)
22. Overall evidence	91 (100.0)	0 (0.0)	0 (0.0)

TREND = Transparent Reporting of Evaluations with Nonrandomized Designs.

**Table 3.** Mapping the TREND Checklist to TIDieR, JBI, and ROBINS-I V2 for Quasi-experimental Studies

Item No.	TREND domain	TREND items	TIDieR	JBI	ROBINS-I V2
1	Title and abstract	Allocation method, structured abstract, target population/sample	1		
2	Introduction	Scientific rationale, theoretical framework	2		Domain 1
3	Methods: participants	Eligibility, recruitment method, and data collection setting		2	Domain 2
4	Methods: interventions	Intervention details and delivery	3~12	4	Domain 3, 4
5	Methods: objectives	Specific objectives and hypotheses		1	Domain 6
6	Methods: outcomes	Outcome definition & measurement		5,6,7,8	Domain 6
7	Methods: sample size	Sample size determination			Domain 1
8	Methods: assignment method	Assignment unit and allocation method			Domain 6
9	Methods: blinding (masking)	Blinding procedures			Domain 6
10	Methods: unit of Analysis	Unit of analysis and statistical adjustment			Domain 7
11	Methods: statistical methods	Statistical methods and software used		9	Domain 1
12	Results: participant flow	Participant flow and protocol deviations			Domain 4, 5
13	Results: recruitment	Recruitment and follow-up periods			
14	Results: baseline data	Baseline characteristics and group comparability		2,3	Domain 1
15	Results: baseline equivalence	Baseline group equivalence and adjustment methods		5	Domain 1
16	Results: numbers analyzed	Analysis denominators and intention-to-treat approach		8	
17	Results: outcomes and estimation	Outcome results, effect sizes, and causal pathway testing		7,8	Domain 7
18	Results: ancillary analyses	Subgroup and exploratory analyses		9	Domain 7
19	Results: adverse events	Adverse events and unintended effects			Domain 7
20	Discussion: interpretation	Interpretation, mechanisms, implementation fidelity, and implications			
21	Discussion: generalizability	Generalizability and contextual considerations			
22	Discussion: overall evidence	Results in context of existing evidence and theory			

TREND = Transparent Reporting of Evaluations with Nonrandomized Designs; TIDieR = Template for Intervention Description and Replication Checklist; JBI = Joanna Briggs Institute Critical Appraisal Checklist for Quasi-Experimental Studies; ROBINS-I V2 = Risk of Bias in Non-Randomized Studies of Interventions, Version 2.

## Data Availability

All data extracted from published articles are available upon reasonable request.

## Appendix 1.

**A1.** Kwak MG, Kim YK, Hong HS. The effects of abdominal breathing on preoperative anxiety and blood pressure of upper and lower limbs surgical patients. Journal of Korean Biological Nursing Science. 2011;13(1):23-28.

**A2.** Lee SY. Effects of aromatherapy hand massage on anxiety, depression, sleep disturbance, and fatigue of the institutionalized elderly. Journal of Korean Biological Nursing Science. 2011;13(1):29-36.

**A3.** Lee JM, Hong HS. The effect of preference music therapy on anxiety and pain of cystoscopy. Journal of Korean Biological Nursing Science. 2011;13(1):44-52.

**A4.** Kang IS, Kim YH. The effect of recorded mother’s voice on the body weight and physiological reactions of premature infants. Journal of Korean Biological Nursing Science. 2011;13(2):94-101.

**A5.** Kim JI, Lee JS. Effects of clinical training on stress, anxiety, and changes in autonomic nervous system in nursing students. Journal of Korean Biological Nursing Science. 2011;13(2):102-108.

**A6.** Yun JY, Lee KE. Effect of nursing information on ICU patient’s environmental stress, anxiety, and comfort. Journal of Korean Biological Nursing Science. 2011;13(2):109-116.

**A7.** Kim YO, Kim JH. Effects of types of music in music therapy on anxiety and vital signs of surgical patients undergoing operation using spinal anesthesia. Journal of Korean Biological Nursing Science. 2011;13(2):149-155.

**A8.** Kim HJ. Comparison of bacterial contamination according to the using period of feeding bag and disinfection methods in neurosurgery intensive care unit patients. Journal of Korean Biological Nursing Science. 2011;13(3):204-210.

**A9.** Lim NY, Jeong HC, Lee SW, Kim WJ. Effect of heat therapy to superficial and deep body temperatures according to applying dry and moist heats on shoulder and thigh. Journal of Korean Biological Nursing Science. 2011;13(3):269-275.

**A10.** Song MS, Kim SK, Yoo YK, Kim HJ, Kim NC. Effects of the aquatic exercise program on body fat, skeletal muscle mass, physical fitness, and depression in elderly women. Journal of Korean Biological Nursing Science. 2011;13(3):276-282.

**A11.** Yu GW, Min S, Ha YJ. The effect of stepbox exercise program on both male and female elderly’s cardiovascular and physiological index. Journal of Korean Biological Nursing Science. 2011;13(3):291-297.

**A12.** Kim EJ, Yoon HS. Influence of midazolam and glycopyrrolate on intra-operative body temperature in abdominal surgical patients. Journal of Korean Biological Nursing Science. 2012;14(1):25-32.

**A13.** Kim DO, Lee HS, Kwon YS. The effects of dance sports program on health promotion in rural women. Journal of Korean Biological Nursing Science. 2012;14(2):84-93. http://doi.org/10.7586/jkbns.2012.14.2.84

**A14.** Choe EY, Jeong SH. The effect of self-efficacy promotion smoking cessation program on the amount of smoking, CO, urine cotinine level, and self-efficacy for adolescent smokers. Journal of Korean Biological Nursing Science. 2012;14(2):103-111. http://doi.org/10.7586/jkbns.2012.14.2.103

**A15.** Kim SH, Lee MH, Kang JH, Jeong SH. The effects of a fluid intake intervention for elders in long-term care hospitals. Journal of Korean Biological Nursing Science. 2012;14(2):139-146. http://doi.org/10.7586/jkbns.2012.14.2.139

**A16.** Park JH, Kim HS. The effect of the hand hygiene education program on hand hygiene knowledge, hand hygiene perception, nasal *Staphylococcus aureus* colonization, and hand hygiene adherence in nursing students. Journal of Korean Biological Nursing Science. 2012;14(3):156-165. http://doi.org/10.7586/jkbns.2012.14.3.156

**A17.** Jeong EJ, Chae YR. The effects of self stretching on shoulder pain and shoulder flexibility of hospital nurses. Journal of Korean Biological Nursing Science. 2012;14(4):268-274. http://doi.org/10.7586/jkbns.2012.14.4.268

**A18.** Kim AL. Effects of structured arm exercise on arteriovenous fistula stenosis in hemodialysis patient. Journal of Korean Biological Nursing Science. 2012;14(4):300-307. http://doi.org/10.7586/jkbns.2012.14.4.300

**A19.** Shin MG, Min S, Jeong SM, Lee YR. The effect of line dance program on oxygen saturation and happiness of elderly. Journal of Korean Biological Nursing Science. 2013;15(1):1-7. http://doi.org/10.7586/jkbns.2013.15.1.1

**A20.** Kim BH, Chang SJ, Choi JS. The development and evaluation of a clinical practice nursing students’ microbiology program based on the mastery learning model. Journal of Korean Biological Nursing Science. 2013;15(2):90-98. http://doi.org/10.7586/jkbns.2013.15.2.90

**A21.** Lee JM, Hong HS. The effect of transcutaneous electrical nerve stimulation on acute pain and beta-endorphins of needle biopsy. Journal of Korean Biological Nursing Science. 2013;15(3):99-106. http://doi.org/10.7586/jkbns.2013.15.3.99

**A22.** Suk YM, Park JW, Jeon MJ, Kim CY. Effect of periodic video education on knowledge about hemodialysis, patient role behavior, and the physiologic index in patients with hemodialysis. Journal of Korean Biological Nursing Science. 2013;15(3):122-132. http://doi.org/10.7586/jkbns.2013.15.3.122

**A23.** Kim JH, Hyun HJ, Ahn MH, Choi EY, Ko GY, Park BS. The effects of the thera band exercise program on bone mineral density and health promotion behaviors in elderly women. Journal of Korean Biological Nursing Science. 2013;15(3):147-153. http://doi.org/10.7586/jkbns.2013.15.3.147

**A24.** Park JE, Kim HS, Hong HS. Shortening of nursing record time about real time transmission effect of blood pressure, blood glucose value based on U-healthcare. Journal of Korean Biological Nursing Science. 2013;15(4):164-172. http://doi.org/10.7586/jkbns.2013.15.4.164

**A25.** Cha MY, Hong HS. Effects on the laughter score, cortisol, and immunoglobulin of laughter therapy in middle aged women. Journal of Korean Biological Nursing Science. 2013;15(4):230-236. http://doi.org/10.7586/jkbns.2013.15.4.230

**A26.** Ahn MN, Ahn HY. The effects of music intervention on pain among critically ill patients with ventilatory support. Journal of Korean Biological Nursing Science. 2013;15(4):247-256. http://doi.org/10.7586/jkbns.2013.15.4.247

**A27.** Paek YW, Min S, Lee BH, Shin MG. Changes in pain, muscle strength, and flexibility according to pinch lift and rubbing manual therapy and stretching application for low back pain. Journal of Korean Biological Nursing Science. 2014;16(1):1-7. http://doi.org/10.7586/jkbns.2014.16.1.1

**A28.** Lee HN, Kim JH. The effect of inter dental brush education on the dental plaque index and the degree of halitosis for elementary school students. Journal of Korean Biological Nursing Science. 2014;16(1):8-16. http://doi.org/10.7586/jkbns.2014.16.1.8

**A29.** Son MA, Lee YM, Jung KA. The effects of a combined exercise program on obesity and metabolic syndrome factors for chronic psychiatric inpatients. Journal of Korean Biological Nursing Science. 2014;16(2):105-112. http://doi.org/10.7586/jkbns.2014.16.2.105

**A30.** Ko SJ, Na YK, Hong HS. Effects of normal saline and essential oil gargling on bacterial colonization in intubated patients for general anesthesia. Journal of Korean Biological Nursing Science. 2014;16(2):123-132. http://doi.org/10.7586/jkbns.2014.16.2.123

**A31.** Jeong SH, Kim NS, Chae S, Lee EJ. Effects of an extreme heat adaptation program in hypertensive patients. Journal of Korean Biological Nursing Science. 2014;16(3):164-172. http://doi.org/10.7586/jkbns.2014.16.3.164

**A32.** Lee HS, Song MR. The effects of a quit smoking program using the web and short message service on exhaled carbon monoxide, self-efficacy, and depression according to nicotine dependency level in undergraduate students. Journal of Korean Biological Nursing Science. 2014;16(3):173-181. http://doi.org/10.7586/jkbns.2014.16.3.173

**A33.** Bae HJ, Kim JH. A study on the effects of ankle pump exercise in reducing lower limbs edema and pain of operating room nurses. Journal of Korean Biological Nursing Science. 2014;16(3):235-243. http://doi.org/10.7586/jkbns.2014.16.3.235

**A34.** Song MR, Kim EK. Effects of eucalyptus aroma therapy on the allergic rhinitis of university students. Journal of Korean Biological Nursing Science. 2014;16(4):300-308. http://doi.org/10.7586/jkbns.2014.16.4.300

**A35.** Shim JH, Choi-Kwon S. The effect of pre-operative patient controlled analgesia education on elderly patients with total knee arthroplasty. Journal of Korean Biological Nursing Science. 2014;16(4):318-325. http://doi.org/10.7586/jkbns.2014.16.4.318

**A36.** Kim MH, Kim JI, Ha E. Effects of aroma-necklace application on perceived stress, symptoms of stress and changes in autonomic nervous system among nursing students in clinical training. Journal of Korean Biological Nursing Science. 2014;16(4):334-341. http://doi.org/10.7586/jkbns.2014.16.4.334

**A37.** Lee YJ, Park SM. Effects of pressure ulcer classification system education program on knowledge and visual discrimination ability of pressure ulcer classification and incontinence-associated dermatitis for hospital nurses. Journal of Korean Biological Nursing Science. 2014;16(4):342-348. http://doi.org/10.7586/jkbns.2014.16.4.342

**A38.** Kim JS, Hyun HJ. Effect of program promoting intention to exercise performance based theory of planned behavior in the elderly. Journal of Korean Biological Nursing Science. 2015;17(1):1-10. https://doi.org/10.7586/jkbns.2015.17.1.1

**A39.** Byun GR, Park JE, Hong HS. The effects of video programs of cardiopulmonary cerebral resuscitation education. Journal of Korean Biological Nursing Science. 2015;17(1):19-27. https://doi.org/10.7586/jkbns.2015.17.1.19

**A40.** Kim NH, Park JY, Jun SE. The effects of case-based learning (CBL) on learning motivation and learning satisfaction of nursing students in a human physiology course. Journal of Korean Biological Nursing Science. 2015;17(1):78-87. https://doi.org/10.7586/jkbns.2015.17.1.78

**A41.** Jo YM, Chae YR, Eom JH. A comparison of different application times of oral care on colonies of microorganisms and oral health status on intubated patients. Journal of Korean Biological Nursing Science. 2015;17(2):97-103. http://doi.org/10.7586/jkbns.2015.17.2.97

**A42.** Kim CS, Jang SH, Cho YY. The effect of laughter therapy on arthralgia, ankylosis, depression, and sleep of elderly housebound women with osteoarthritis. Journal of Korean Biological Nursing Science. 2015;17(2):123-131. http://doi.org/10.7586/jkbns.2015.17.2.123

**A43.** Lee JS, Chung YH. Effect of acupressure massage on temperatures of acupoints, severity of facial paralysis, subjective symptoms, and depression in Bell’s palsy patients. Journal of Korean Biological Nursing Science. 2015;17(2):140-149. http://doi.org/10.7586/jkbns.2015.17.2.140

**A44.** Son HJ, Hong HS. The effects of S-solution and A-solution on oral health in preschool children. Journal of Korean Biological Nursing Science. 2015;17(2):150-158. http://doi.org/10.7586/jkbns.2015.17.2.150

**A45.** Kil MS, Lee MH, Lee YM. Effects of a recreation therapy program on mental health and heart rate variability in burn rehabilitation patients. Journal of Korean Biological Nursing Science. 2015;17(2):179-187. http://doi.org/10.7586/jkbns.2015.17.2.179

**A46.** Chae KS. Effects of laughing and music therapy on depression and activities of the autonomic nervous system in the elderly with dementia. Journal of Korean Biological Nursing Science. 2015;17(3):245-252. http://doi.org/10.7586/jkbns.2015.17.3.245

**A47.** Hwang MS, Kim JH. Effects of PCA (patient controlled analgesics) education program including practicum on post-op pain of gynecologic laparoscopic surgery patients. Journal of Korean Biological Nursing Science. 2015;17(3):253-261. http://doi.org/10.7586/jkbns.2015.17.3.253

**A48.** Yoo KH. Effects of music therapy on the heart rate and respiration rate in premature infants. Journal of Korean Biological Nursing Science. 2015;17(3):271-276. http://doi.org/10.7586/jkbns.2015.17.3.271

**A49.** Lim JS, Jo HS. Effects of fluid therapy education program for aged stroke patients. Journal of Korean Biological Nursing Science. 2015;17(3):277-285. http://doi.org/10.7586/jkbns.2015.17.3.277

**A50.** Hwang YH, Yi MS. Evaluation of an individualized education before discharge and follow-up telephone consultation on self-efficacy for kidney transplant patients. Journal of Korean Biological Nursing Science. 2015;17(4):331-340. http://doi.org/10.7586/jkbns.2015.17.4.331

**A51.** Son YL, Yoo MS. Effects of a footbath program on heart rate variability, blood pressure, body temperature, and fatigue in stroke patients. Journal of Korean Biological Nursing Science. 2016;18(1):51-59. http://doi.org/10.7586/jkbns.2016.18.1.51

**A52.** Kim JY, Na YK, Hong HS. The effects of a secondary stroke prevention program on the health risk indicators and self-care compliance of stroke patients. Journal of Korean Biological Nursing Science. 2016;18(2):69-77. http://doi.org/10.7586/jkbns.2016.18.2.69

**A53.** Kim JY, Cheon EY. The effects of a self-care management program for patients with diabetic foot ulcers. Journal of Korean Biological Nursing Science. 2016;18(2):78-86. http://doi.org/10.7586/jkbns.2016.18.2.78

**A54.** Kim JH, Hyun HJ, Kang SY, Nam HR, Shin MJ, Lee HJ, et al. Effects of hand massage with nail art on depression, self-esteem, and vital signs of elderly women living in a nursing home. Journal of Korean Biological Nursing Science. 2016;18(3):169-175. http://doi.org/10.7586/jkbns.2016.18.3.169

**A55.** Lee MH, Park MS. The effect of case-based learning (CBL) on critical thinking disposition, communication ability, problem solving ability and self-directed learning ability of nursing students in pathophysiology course. Journal of Korean Biological Nursing Science. 2016;18(3):176-184. http://doi.org/10.7586/jkbns.2016.18.3.176

**A56.** Lee KO. Effects of calcium/vitamin D intake and taekkyeon exercise on the elderly’s frailty. Journal of Korean Biological Nursing Science. 2016;18(3):185-191. http://doi.org/10.7586/jkbns.2016.18.3.185

**A57.** Jang IS, Park SM. The effect of denture care skills education program on denture self-care, denture satisfaction, and oral health-related quality of life (OHIP-14) among the elderly. Journal of Korean Biological Nursing Science. 2016;18(4):239-246. https://doi.org/10.7586/jkbns.2016.18.4.239

**A58.** Shin EJ, Jung JS, Choi SH, Huh IY. Impact of central line insertion bundle on the adherence of bundle and central line-associated bloodstream infections in the operating room. Journal of Korean Biological Nursing Science. 2016;18(4):257-263. https://doi.org/10.7586/jkbns.2016.18.4.257

**A59.** Lee SY, Lee KS. Effects of information provision on anxiety, blood pressure, and pulse in cerebral angiography clients. Journal of Korean Biological Nursing Science. 2016;18(4):280-287. https://doi.org/10.7586/jkbns.2016.18.4.280

**A60.** Lim KC, Chun MH. The effects of a functional game (Rejuvenescent Village) for older Koreans’ cognitive function, instrumental activities of daily living, depression, and life satisfaction. Journal of Korean Biological Nursing Science. 2016;18(4):296-304. https://doi.org/10.7586/jkbns.2016.18.4.296

**A61.** Kim YM, Kim MY, Seo YH. The effects of an intensive education program on hospital infection control on nursing students’ knowledge, attitude, and confidence in infection control. Journal of Korean Biological Nursing Science. 2016;18(4):318-326. https://doi.org/10.7586/jkbns.2016.18.4.318

**A62.** Kim SS, Jo HS, Kang MS. Retention effects of dietary education program on diet knowledge, diet self-care compliance, physiologic indices for hemodialysis patients. Journal of Korean Biological Nursing Science. 2017;19(2):51-59. https://doi.org/10.7586/jkbns.2017.19.2.51

**A63.** Oh IO, Yoo JY, Oh EG. Development and evaluation of an evidence-based nursing protocol for postoperative nausea and vomiting. Journal of Korean Biological Nursing Science. 2017;19(2):86-97. https://doi.org/10.7586/jkbns.2017.19.2.86

**A64.** Choi SH, Choi-Kwon S, Kwak CS, Lee HY. The effects of Korean DASH diet education program on oxidative stress, antioxidant capacity, and serum homocysteine level among elderly Korean women. Journal of Korean Biological Nursing Science. 2017;19(3):141-150. https://doi.org/10.7586/jkbns.2017.19.3.141

**A65.** Kang SY, Kim JH. Comparison of the effects on sleep and vital signs of the elderly between the hand bath group and the foot bath group. Journal of Korean Biological Nursing Science. 2017;19(3):151-157. https://doi.org/10.7586/jkbns.2017.19.3.151

**A66.** Choo HS, Kim JH. The effects of gratitude enhancement program on psycho-social and physical health of chronic schizophrenia. Journal of Korean Biological Nursing Science. 2017;19(3):158-169. https://doi.org/10.7586/jkbns.2017.19.3.158

**A67.** Song HJ, Park HK, Jwa SH, Moon SH, Kim SH, Shin JY, et al. The effectiveness of community-based muscle and joint self-management program for older adults. Journal of Korean Biological Nursing Science. 2017;19(3):191-197. https://doi.org/10.7586/jkbns.2017.19.3.191

**A68.** Park EH, Chae YR. The effects of self-leadership reinforcement program for hospital nurses. Journal of Korean Biological Nursing Science. 2018;20(2):132-140. https://doi.org/10.7586/jkbns.2018.20.2.132

**A69.** Ju HJ, Jeon MY. Effects of walking program with dance on gait, cognition, and risk of falls of elderly with dementia in a long-term care hospital. Journal of Korean Biological Nursing Science. 2018;20(3):141-149. https://doi.org/10.7586/jkbns.2018.20.3.141

**A70.** Kim SS, Choi YS. Effect of individual low sodium dialysate on blood pressure, interdialytic weight gain, thirst, and intradialytic discomfort in end-stage renal disease patients. Journal of Korean Biological Nursing Science. 2019;21(3):169-177. https://doi.org/10.7586/jkbns.2019.21.3.169

**A71.** Choi JY, Jo HS. Effects of the team approach rehabilitation program on balance, gait, and muscle strength of lower extremities for elderly patients with Parkinson’s disease. Journal of Korean Biological Nursing Science. 2019;21(3):199-206. https://doi.org/10.7586/jkbns.2019.21.3.199

**A72.** Park HS, Shin NY. Comparison of effects of different acupressure methods on nausea, vomiting, and anorexia for breast cancer patients: among patients undergoing chemotherapy. Journal of Korean Biological Nursing Science. 2020;22(2):102-110. https://doi.org/10.7586/jkbns.2020.22.2.102

**A73.** Park YJ, Kim N. The effects of seogeum therapy on nasal eosinophil, nasal symptoms, and rhinitis related quality of life in college students with allergic rhinitis. Journal of Korean Biological Nursing Science. 2020;22(2):127-138. https://doi.org/10.7586/jkbns.2020.22.2.127

**A74.** Back JY, Jun SE. The effects of a motivation-enhanced self-management program for female college students with irritable bowel syndrome. Journal of Korean Biological Nursing Science. 2020;22(2):148-156. https://doi.org/10.7586/jkbns.2020.22.2.148

**A75.** Kang KS, Choi H. The effects of compliance and self efficacy on nursing education program for pneumonia patient. Journal of Korean Biological Nursing Science. 2020;22(3):184-191. https://doi.org/10.7586/jkbns.2020.22.3.184

**A76.** No HJ, Eun Y, Park HW, Cheon MH. The effects of nebulizer therapy with normal saline on postoperative thirst and sore throat. Journal of Korean Biological Nursing Science. 2020;22(2):204-212. https://doi.org/10.7586/jkbns.2020.22.3.204

**A77.** Lee SH, Choi JS. The development and evaluation of a motivation-strengthening obesity management program for obese subjects with visual impairment. Journal of Korean Biological Nursing Science. 2020;22(4):232-248. https://doi.org/10.7586/jkbns.2020.22.4.232

**A78.** Kim BH, Jeong YH. Effects of a case-based sepsis education program for general ward nurses on knowledge, accuracy of sepsis assessment, and self-efficacy. Journal of Korean Biological Nursing Science. 2020;22(4):260-270. https://doi.org/10.7586/jkbns.2020.22.4.260

**A79.** Lee YS, Lee BJ, Ha CY, Jeon MY. Effects of an educational program based on mobile SMS and counseling for colonoscopy in the elderly. Journal of Korean Biological Nursing Science. 2021;23(1):64-71. https://doi.org/10.7586/jkbns.2021.23.1.64

**A80.** Kang SY, Chae YR. Effect of antioxidant improvement program with health contract on antioxidant indicators and body composition in female college students. Journal of Korean Biological Nursing Science. 2021;23(3):188-198. https://doi.org/10.7586/jkbns.2021.23.3.188

**A81.** Choi EA, Jeon MY. Effects of family conflict mitigation programs by watching documentaries on conflicts, autonomic nerve activation, and happiness of the elderly in long-term care hospitals. Journal of Korean Biological Nursing Science. 2021;23(3):237-246. https://doi.org/10.7586/jkbns.2021.23.3.237

**A82.** Kang HY, Im HG, Chae YR. Effect of life-oriented forest healing program in urban forest on body composition, psychological state, and quality of life of adults over 40 years of age. Journal of Korean Biological Nursing Science. 2022;24(1):36-45. https://doi.org/10.7586/jkbns.2022.24.1.36

**A83.** Cho MJ, Jeong JS, Kim YH. Effect of multifaceted interventions for ward nurses on the storage, conditions, and transportation of specimens for microbial culture. Journal of Korean Biological Nursing Science. 2022;24(2):95-103. https://doi.org/10.7586/jkbns.2022.24.2.95

**A84.** Chae YR, Lee SH, Kim SY, Choi JK. Efficacy of forest-thermal combined therapy for anxiety and stress among smoking-cessation attempters. Journal of Korean Biological Nursing Science. 2022;24(4):227-234. https://doi.org/10.7586/jkbns.2022.24.4.227

**A85.** Jung MR, Jeong E, Lee CG. Development and effectiveness of a cognitive enhancement program based on a mobile application for preventing dementia: a study focusing on older adults who use senior citizen centers. Journal of Korean Biological Nursing Science. 2023;25(1):113-122. https://doi.org/10.7586/jkbns.23.0005

**A86.** Nam HW, Jun SE. Development and evaluation of a mobile app-based musculoskeletal exercise program for operating room nurses. Journal of Korean Biological Nursing Science. 2023;25(3):215-227. https://doi.org/10.7586/jkbns.23.0008

**A87.** Seo WK, Kim HJ. Effects of a video-based enteral nutrition education program using QR codes for intensive care unit nurses: a quasi-experimental study. Journal of Korean Biological Nursing Science. 2024;26(1):16-25. https://doi.org/10.7586/jkbns.24.001

**A88.** Shin YK, Kim MY. Effects of self-care intervention using a mobile instant messenger on hemodialysis patient’s knowledge, self-efficacy, self-care behavior, and physiological index. Journal of Korean Biological Nursing Science. 2024;26(2):123-135. https://doi.org/10.7586/jkbns.24.004

**A89.** Chae YR, Park SY, Kang SY, Kang HY, Lee SH, Jo YM, et al. Effects of a forest therapy camp on cancer survivors’ stress, mood, and natural killer cells in Korea. Journal of Korean Biological Nursing Science. 2024;26(3):185-194. https://doi.org/10.7586/jkbns.24.011

**A90.** Kong JH, Lee S, Jeon MY. Effects of the healing movie programs on post-traumatic stress syndrome, resilience, and cognitive emotional control strategies of Korean cancer survivors: a non-equivalent control group pretest-posttest design. Journal of Korean Biological Nursing Science. 2024;26(3):195-205. https://doi.org/10.7586/jkbns.24.015

**A91.** Kim SH, Choi Y. Improving clinical reasoning competency and communication skills using virtual simulation-based learning focused on a pathophysiological approach in Korea: a quasi-experimental study. Journal of Korean Biological Nursing Science. 2024;26(4):363-372. https://doi.org/10.7586/jkbns.24.026
